# Genotypic and Phenotypic Insights on 11 Novel Variants in the *ABCA4* Gene

**DOI:** 10.3390/genes17070728

**Published:** 2026-06-23

**Authors:** Saoud Al-Khuzaei, Jing Yu, Suzanne Broadgate, Morag Shanks, Penny Clouston, Robert E. MacLaren, Peter Charbel Issa, Stephanie Halford, Samantha R. De Silva, Susan M. Downes

**Affiliations:** 1 Oxford Eye Hospital, John Radcliffe Hospital, Oxford University Hospitals NHS Foundation Trust, Oxford OX3 9DU, UK; 2 Nuffield Laboratory of Ophthalmology, Nuffield Department of Clinical Neuroscience, University of Oxford, Level 6 John Radcliffe Hospital, Headley Way, Oxford OX3 9DU, UK; 3 Oxford Medical Genetics Laboratories, Oxford University Hospitals NHS Foundation Trust, Oxford OX3 7LE, UK; 4 Department of Ophthalmology, Technical University, 80333 Munich, Germany

**Keywords:** *ABCA4*, novel variants, Stargardt disease, panel-based NGS, *ABCA4* retinopathy

## Abstract

**Objectives**: The aim of this study was to report novel *ABCA4* variants detected in a cohort of 259 patients with *ABCA4* retinopathy with the intention of improving the diagnostic accuracy for *ABCA4* retinopathy and expanding its genetic spectrum. **Methods**: We retrospectively reviewed 259 patients with *ABCA4* retinopathy, comprising 190 patients from the Oxford Cohort and 69 patients from other centres with a clinical diagnosis of *ABCA4* retinopathy who were referred for genetic testing. Patients with a phenotype consistent with *ABCA4* retinopathy who had a novel *ABCA4* variant were included. Phenotyping in the Oxford Cohort included clinical evaluation, retinal imaging, and electrodiagnostic testing. Genetic testing was performed using next-generation sequencing (NGS) and Sanger sequencing. In silico analysis was used to investigate the pathogenicity of novel variants. **Results**: Eleven novel variants were detected in 12/259 patients, with one variant detected in two unrelated patients. These variants included three missense, four truncating, three splice-site variants, and one exon deletion. The variants were distributed across eight exons and three introns of *ABCA4*. In silico analysis and phenotype correlation supported the potential pathogenicity of the novel variants. Phenotypes ranged from mild isolated flecks with preserved retinal architecture to extensive chorioretinal degeneration. **Conclusions**: Despite over 2200 *ABCA4* variants being reported to date, a further 11 novel *ABCA4* variants were identified in 259 patients using NGS panel-based sequencing and MLPA. The variants were located across the whole *ABCA4* gene, emphasising the necessity to sequence the whole gene. Our reporting of these variants expands the known genetic spectrum of *ABCA4* retinopathy, contributing to accurate diagnosis in this patient group and the identification of suitable patients for recruitment to potential therapeutic interventions.

## 1. Introduction

Stargardt disease (STGD1, OMIM# 248200) is the most common childhood onset macular dystrophy and the most prevalent inherited retinal disease, with a worldwide prevalence estimated to be 1/6578, with a carrier rate of 2.5% in the European subpopulation [1]. STGD1 is an autosomal recessive condition caused by pathogenic variants in the *ABCA4* gene [2]. Characteristic features include the presence of flecks, increased lipofuscin levels, macular atrophy, and sparing of the peripapillary region [3,4]. However, the disease is phenotypically heterogeneous, and a number of features described in this condition are encompassed by the term *ABCA4* retinopathies, representing a phenotypic spectrum which includes: bull’s eye maculopathy [5,6], cone-rod dystrophy [7], choriocapillaris atrophy [8], and rapid onset chorioretinal atrophy [9]. This impacts a clinician’s ability to diagnose this condition and emphasises the need for molecular genetic testing to accurately diagnose *ABCA4* retinopathy patients.

Providing a molecularly confirmed diagnosis is further complicated by the highly polymorphic nature of the *ABCA4* gene, and to date, over 2200 *ABCA4* variants have been reported (www.lovd.nl/ABCA4, accessed on 10 April 2026). The majority of *ABCA4* variants are missense changes, which can make it difficult to determine whether the identified variants are truly causative, and this raises the need for genotypic and phenotypic characterisation in large patient cohorts to fully investigate the effects of suspected disease-causing variants. Documenting novel variants is important since obtaining a molecularly confirmed diagnosis is essential for recruitment to clinical trials and consideration of future therapeutic interventions. In this study, we report the genotypes and phenotypes of 12 patients in whom novel variants in *ABCA4* were identified in a cohort of 259 *ABCA4* retinopathy patients who had their genetic testing at the Oxford Clinical Genetics laboratory.

## 2. Materials and Methods

### 2.1. Patient Recruitment

Patients with at least one variant in *ABCA4* detected between November 2009 and October 2021 were identified by the Oxford University Hospitals Medical Genetics Laboratory. This included patients who were reviewed and received care at the Oxford Eye Hospital, designated as the “Oxford Cohort” (190 patients), and those referred for genetic testing from other centres within the United Kingdom and abroad (69 patients), accounting for 259 patients. All patients included were described as having an *ABCA4* retinopathy phenotype and had at least one *ABCA4* variant. Complex alleles were investigated using familial segregation studies, as available, and, in cases where segregation was not possible, the literature was reviewed to identify if patients’ detected variants were previously reported to be in a complex allele. Segregation analysis was available for only 92 families and confirmed biallelic status in 93/259 patients in this study. The study adhered to the tenets of the Declaration of Helsinki and was approved by the Central Oxford Research Ethics Committee and the Research and Development Department of the Oxford Radcliffe Hospitals NHS Trust (RetGene 08/H0302/96).

### 2.2. Phenotypic Analysis

All patients included in the Oxford Cohort were reviewed in a specialist Ophthalmic Genetics Clinic at the Oxford Eye Hospital, where a detailed medical history was taken, and a clinical eye examination was performed. Best corrected visual acuity (BCVA) was recorded either on Snellen or LogMAR charts, and all Snellen measurements were converted to LogMAR for the purpose of statistical analysis. A BCVA of counting fingers (CF) and hand movements (HM) was converted to 2.5 logMAR and 3.0 logMAR, respectively. Retinal imaging (after pupil dilation with tropicamide 1% and phenylephrine 2.5%) included colour fundus photography (TopCon), wide-field retinal imaging (Optomap A10022; Optos Ltd., Dunfermline, UK), short-wavelength fundus autofluorescence (Spectralis; Heidelberg Engineering, Heidelberg, Germany) (488 nm), and spectral-domain optical coherence tomography (SD-OCT) (SD-OCT; Spectralis, Heidelberg Engineering, Heidelberg, Germany). Electrodiagnostic testing, where indicated, was carried out according to the International Society for Clinical Electrophysiology of Vision (ISCEV) [10]. The 69 patients from other centres were all referred with an *ABCA4* retinopathy clinical diagnosis.

### 2.3. Genetic Testing and Mutation Analysis

DNA was extracted from peripheral venous blood samples. Sequencing was performed according to the most appropriate method available at the Oxford Medical Genetics Laboratory at the time at which the patients presented. Enrichment of the *ABCA4* gene was achieved using a customised HaloPlex enrichment system kit (Agilent Technologies, Santa Clara, CA, USA) designed to capture the coding exons and 10 bp flanking introns [11]. Between 2019 and 2022, samples were prepared using Twist Human Core Exome [12]. Next-generation sequencing (NGS) was carried out using an Illumina MiSeq instrument (Illumina, San Diego, CA, USA), using a MiSeq v3 kit as per the manufacturer’s instructions [11]. Multiplex ligation-dependent probe amplification (MLPA) analysis was also carried out to identify deletions and duplications. MLPA was run as standard for any patient referred with a clinical diagnosis of STGD1 or when specific sequencing for *ABCA4* was requested in patients. This was performed for patients who originally had Sanger sequencing of *ABCA4* (pre 2013) or Haloplex (2013–2019). For Haloplex-based testing, the number of reads mapped to the exon was used to calculate the dosage quotient (DQ) and internal reference exons were used if they had an average coverage >50 and up to 60 DQs were calculated for each exon. CNV calling was carried out as variants were identified using a custom-designed application called Dosage Quotient for Massive Parallel Sequencing (DosQuoMPS), and the CNVs were confirmed using MLPA. However, MLPA analysis was not performed on patients who had sequencing using the TWIST protocol, unless it was required to confirm a possible CNV call from the bioinformatics pipeline [11]. More detailed information on sequencing in our cohort is available in a previous publication by our group, published by Mital et al. [11].

Chromosome 1 position was based on the GRCh37/hg19 build, and nucleotide and protein numbering were based on the *ABCA4* transcript NC_000001.10. Protein alignment was based on the protein transcript NP_000341.2. In silico analysis was performed using three prediction methods to predict the pathogenicity of the identified variants. Missense variants were investigated using PolyPhen2 (http://genetics.bwh.harvard.edu/pph2/, accessed on 10 April 2026) [13], Sorting Intolerant from Tolerance (SIFT) (http://sift.jcvi.org/, accessed on 10 April 2026) [14], and MutationTaster (http://www.mutationtaster.org/, accessed on 10 April 2026 ) [15]. The combined annotation-dependent duplication (CADD) score for all variants was also calculated (https://cadd.gs.washington.edu, accessed on 10 April 2026) [16]. Splice defects were investigated using SpliceAI (https://spliceailookup.broadinstitute.org, accessed on 10 April 2026) [17]. The Exome Aggregation Consortium (ExAC) and gnomAD databases (https://gnomad.broadinstitute.org, accessed on 10 April 2026) were used to check variant frequency. The LOVD (www.lovd.nl/ABCA4, accessed on 10 April 2026) and ClinVar (https://www.ncbi.nlm.nih.gov/clinvar/, accessed on 10 April 2026) databases were reviewed to investigate whether the detected variants were previously reported as pathogenic in other patients with *ABCA4* retinopathy. Variants were considered novel if they were not previously published in association with a clear, detailed phenotype of *ABCA4* retinopathy. Of note, the c.365_366insCA p.(Gly123fs*32), c.1100-2A>T, c.4936G>T p.(Asp1646Tyr), and c.1855A>T p.(Ile619Phe) were included as being novel in this study despite being reported in ClinVar because they had not been clearly shown to be linked to a STGD1 phenotype.

The evolutionary conservation of novel missense variants was investigated by aligning amino acid sequences from different species using Clustal Omega (https://www.ebi.ac.uk/Tools/msa/clustalo/, accessed on 10 April 2026). Accession numbers were: human (*Homo sapiens*) NP_000341.2; chimpanzee (*Pan troglodytes*) XP_009423955.3; cow (*Bos taurus*) NP_776646.1; rat (*Rattus norvegicus*) NP_001101191.1; mouse (*Mus musculus*) NP_031404.1; chicken (*Gallus gallus*) XP_015146189.1; frog (*Xenopus tropicalis*) NP_001011033.1; zebrafish (*Danio rerio*) ENSDARP00000123162. Amino acid residues were considered highly conserved if they were preserved across all species or were only different in one species of either fish or reptiles, moderately conserved if they were different in 2 to 5 species, and not conserved if they were different in more than 5 species or at least one primate. The criteria for determining conservation were based on previously described methodology [18].

All variants were classified according to the American College of Medical Genetics and Genomics (ACMG) standards. The Exome Aggregation Consortium (ExAC) and GnomaAD databases (https://gnomad.broadinstitute.org, accessed on 10 April 2026) were used to check variant frequency. Variants with a minor allele frequency of >0.1% were excluded.

Genotypes of patients with biallelic *ABCA4* variants were classified based on the three genotype groups reported in the ProgStar study; Genotype A patients carried two or more severe/null variants, Genotype B patients carried one severe/null variant and one missense or in-frame insertion/deletion, and Genotype C patients carried two or more missense or in-frame insertion/deletion variants (see Table 1). For the purpose of this variant grading tool, variants were considered to be severe if they produced a truncated protein, were splice-site variants, or were synonymous/missense variants that have been shown to affect splicing in the literature [19].

## 3. Results

Two hundred and fifty-nine patients (190 from the Oxford Eye Hospital and 69 referred from other centres) that met our inclusion criteria for a diagnosis of *ABCA4* retinopathy were identified. Within this group of patients, 11 novel variants were identified in 12 biallelic patients (4 males and 8 females). This was from a cohort of 190 patients in whom 171 different *ABCA4* variants were identified. Segregation studies were only available for three of the families with novel variants.

**Figure 1:**
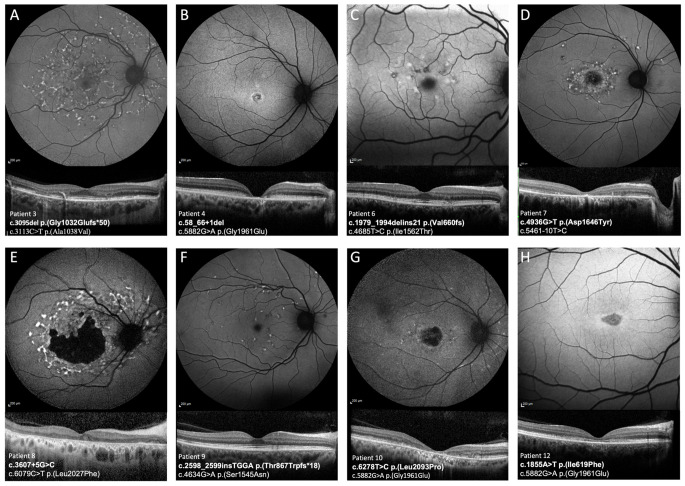
Retinal imaging of patients with novel variants. (**A**–**H**) show fundus autofluorescence (FAF) (upper panel) and optical coherence tomography (OCT) (lower panel) imaging of the right eyes in patients with novel variants. Novel variants are shown in bold. (**A**) In patient 3, FAF imaging showed flecks in the posterior pole, and OCT imaging showed relative preservation of the fovea with a hyper-reflective temporal perifoveal lesion that corresponded to flecks and temporal disruption of the ellipsoid zone (EZ). (**B**) In patient 4, FAF imaging showed reduced foveal AF surrounded by a ring of raised AF and OCT imaging showed central macular atrophy. (**C**) In patient 6, FAF imaging showed perifoveal flecks, and OCT imaging showed relatively preserved central retinal architecture with material arising from the RPE (corresponding to the flecks on FAF), causing elevation of the EZ. (**D**) In patient 7, FAF imaging showed a central reduction in AF signal surrounded by an area of increased AF associated with flecks and flecks in the superior aspect of the posterior pole. OCT imaging showed macular atrophy. (**E**) In patient 8, FAF imaging showed a central reduction in AF signal corresponding to macular atrophy and flecks within the posterior pole, and OCT imaging showed macular atrophy. (**F**) In patient 9, FAF imaging showed flecks in the posterior pole, and OCT imaging showed relatively preserved retinal architecture. (**G**) In patient 10, FAF imaging showed a central reduction in AF signal surrounded by a ring of increased AF and flecks in the posterior pole, and OCT imaging showed macular atrophy. (**H**) In patient 12, FAF imaging showed decreased foveal AF surrounded by a ring of raised AF and OCT imaging showed an foveal optical gap with loss of the EZ.

Phenotypic data were available for the eight patients with novel variants in the Oxford Cohort; no further data were available other than the clinical diagnosis for the external referrals. The age of onset was available for six patients, and there were two asymptomatic patients who were incidentally identified to have flecks during routine examination (see Table 2). Mean age of onset was 36.3 +/− 9.2 years, ranging from 27 to 56 years. Information on symptoms was available for eight patients; the most frequently reported symptom was blurred central vision in 5/8 patients, and other symptoms included photophobia in 1/8, delayed dark adaptation in 2/8, abnormal colour vision in 1/8, and photopsia in 1/8 (see Table 2). The mean baseline visual acuities were 0.56 +/− 0.55 logMAR for the right eye and 0.61 +/−0.74 logMAR for the left eye. Multi-modal imaging revealed a range of phenotypes from flecks only with preserved retinal architecture to extensive chorioretinal degeneration. Of note, patient 8 was initially incorrectly diagnosed with AMD at age 62 before being reviewed in the Ophthalmic Genetics Clinic. The phenotypes on FAF and OCT imaging are illustrated in Figure 1, and detailed information on the clinical characteristics and EDT results is summarised in Table 2, and their genetic test results are summarised in Table 3.

**Figure 2:**
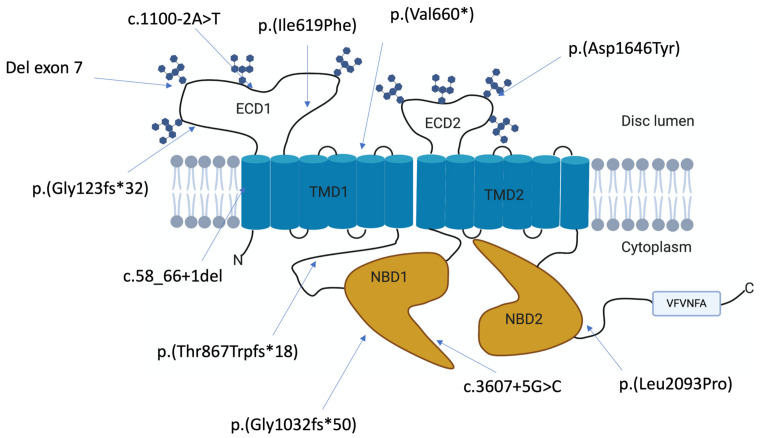
Topological model of the ABCA4 protein showing the distribution of 11 novel variants. Schematic diagram of the ABCA4 protein showing the location of novel variants. The novel variants are distributed across the entirety of the ABCA4 protein. Adapted from [21,22] and created with BioRender.com. ECD: exocytoplasmic domain; NBD: nucleotide-binding domain; TMD: transmembrane domain.

The novel variants included four truncating, three splice-site, three missense variants and one exon deletion. One novel splicing variant, c.3607+5G>C, was identified in 2 unrelated patients (Table 2). These variants were distributed in eight exons and three introns (Table 4). Analysis of the location of the missense variants within the ABCA4 protein revealed that one was located in exocytoplasmic domain 1 (ECD1), one in ECD2, and one in nucleotide-binding domain 2 (NBD2). The novel variants and their corresponding positions along the ABCA4 protein are shown in Figure 2.

In silico analysis of all novel variants is summarised in Table 4. Truncating variants were all considered to be pathogenic since they were expected to result in nonsense-mediated decay (NMD). SpliceAI predicted a splicing defect for c.58_66+1del, c.1100-2A>T, and c.3607+5G>C. Segregation analysis in patient 11 showed that the c.3607+5G>C variant was in *trans* with c.3210_3211dup p.(Ser1071Cysfs*13), as it was detected in her unaffected carrier mother, and c.3607+5G>C was also detected in patient 8, who was unrelated to patient 11; however, segregation analysis was not available for patient 8. This variant was classified as a VUS based on the ACMG criteria.

**Figure 3:**
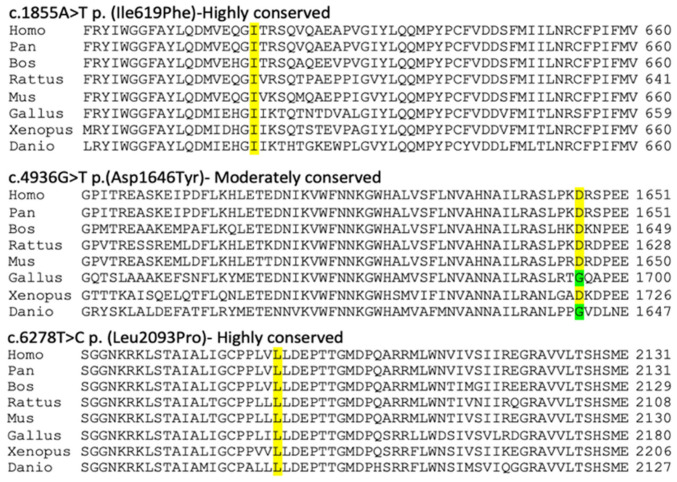
Sequence alignment of ABCA4 across species for novel variants in *ABCA4* retinopathy patients. Assessment of evolutionary conservation of three novel missense variants detected in the 259 patients showing these affect two highly conserved amino acid residues p.(Ile619Phe), and p.(Leu2093Pro), and one moderately conserved amino acid residue p.(Asp1646Tyr). The preserved amino acids are highlighted in yellow and different amino acids are highlighted in green.

Of the three novel missense variants, splicing defects were not predicted in any, but two of these were predicted to be pathogenic by at least two out of three programmes (SIFT, PolyPhen2, MutationTaster). Analysis of these variants using Clustal Omega (https://www.ebi.ac.uk/Tools/msa/clustalo/, accessed on 10 April 2026) showed that the two variants p.(Ile619Phe) and p.(Leu2093Pro) were completely conserved across species. The p.(Asp1646Tyr) variant was moderately conserved across eight different species (Figure 3). There were two siblings who were homozygous for a deletion in exon 7, which was detected using MLPA testing.

## 4. Discussion

Diagnosing *ABCA4* retinopathy can be challenging due to its highly variable phenotype, which makes genetic testing an important aspect of patient care. This study contributes to this endeavour by providing detailed genotype data, together with clinical phenotypes in 12 patients in whom 11 novel variants were detected.

We observed that the novel variants were located across the entire *ABCA4* gene, which is consistent with the current literature on the lack of mutation hot spots in *ABCA4* [23,24], and accounted for 11/171 (6.4%) of the different *ABCA4* variants detected in a cohort of 259 patients which included 190 patients from the Oxford Eye Hospital and 69 patients referred for genetic testing from other centres. Previous reports in large cohorts have shown a higher percentage of novel *ABCA4* variants, for example, 20.4% in ProgStar [19], 33% in German [25], 21.9% in Spanish [26], and 23.3–62.5% in Chinese *ABCA4* retinopathy cohorts, which is a higher proportion of novel variants compared to our cohort [27,28]. The difference in this proportion of novel variants could be attributed to ethnicity: ProgStar recruited from nine centres in four different countries, Fujinami, Strauss, Chiang, Audo, Bernstein, Birch, Bomotti, Cideciyan, Ervin, Marino, Sahel, Mohand-Said, Sunness, Traboulsi, West, Wojciechowski, Zrenner, Michaelides and Scholl [19], and a different *ABCA4* genetic landscape is known from Chinese studies [27,28,29,30]. Moreover, the Oxford Cohort primarily comprised European patients, and given that most *ABCA4* studies have been conducted in patients of this ethnicity, the majority of variants in this population might already be known [31]. Another explanation for the lower percentage of novel variants identified in our study patients might be that a significant proportion of variants have already been detected by improved technology and bioinformatics, which have increased detection rates.

Of the 11 novel variants, four were truncating, three were missense, three affected splice sites, and one was a structural variant. Determining the pathogenicity of missense variants can be complex, which leads to a significant proportion of these variants being classified as a variant of uncertain significance (VUS). The potential pathogenicity of these three variants was supported by in silico analysis predictions. Indeed, the c.1855A>T p.(Ile619Phe) variant Phenylalanine has a larger side chain with an aromatic benzene ring, potentially disrupting the local hydrophobic environment and protein conformation, thus interfering with the TM2 domain structure and overall protein stability. The LOVD contains the c.1856T>A p.(Ile619Asn) variant of uncertain significance and the c.1851_1860del p.(Ile619Alafs*27) pathogenic variant involving the same amino acid. The c.4936G>T p.(Asp1646Tyr) variant results in a change from the negatively charged aspartate to the larger, uncharged, and aromatic tyrosine in the ECD2 domain. This substitution could potentially alter the local charge distribution and hydrogen binding patterns, thus impacting the protein’s interaction with other molecules. The introduction of the bulkier aromatic side chain could also affect the structural stability of the protein. In c.6278T>C p.(Leu2093Pro), leucine is substituted for proline, which is smaller, more flexible, more polar, and introduces a rigid cyclic structure. This rigid structure can potentially disrupt secondary structures such as alpha helices, significantly affecting the local protein structure and potentially leading to misfolding or altered interactions within NBD2.

The detection of a deletion of exon 7 using MLPA sequencing highlighted the need to investigate structural variants, even though they are currently thought to only account for approximately 1% of pathogenic *ABCA4* variants [23]. Complementing this, Khan et al. found that single-molecule molecular inversion probes (sm-MIPs) based sequencing successfully identified 11 novel structural variants in their *ABCA4* retinopathy patients, where initially only one *ABCA4* variant was detected, thus establishing sm-MIPs as an effective technique for detecting structural variants as small as 411 bp [32].

This study had several limitations that were primarily related to its retrospective design and reliance on available data. Complete phenotypic data were not available for all patients, meaning that information on demographics, clinical characteristics, and retinal imaging was incomplete for some patients. Familial segregation was not available for a significant proportion of patients. Additionally, the use of NGS-based sequencing means that pathogenic deep intronic variants would not be detected, and the pathogenicity of variants is based on in silico analysis programmes. The retrospective nature of this study meant that genetic testing techniques were not uniform; for example, MLPA was not routinely offered, which may have led to underdetection of structural variants. All of which are frequently encountered in genotype–phenotype correlation studies in IRD.

## 5. Conclusions

In conclusion, we detected 11 novel *ABCA4* variants in 12 patients with an *ABCA4* retinopathy from 12 unrelated families. The pathogenicity of missense and splice-site variants was investigated using in silico analysis, segregation studies (where available), and the association with the phenotype consistent with *ABCA4* retinopathy. Based on the ACMG criteria, seven were classified as pathogenic and four as VUS. The variants were located across the entire *ABCA4* gene, which is consistent with a lack of mutation hot spots in *ABCA4* and emphasises the need to sequence the whole *ABCA4* gene. MLPA analysis identified an exon deletion, which demonstrates the need to consider structural variants when sequencing *ABCA4*. Our study reports novel ABCA4 variants with varying levels of evidence for their pathogenicity, which contributes to expanding the spectrum of known pathogenic variants in *ABCA4.* This will aid in the accurate diagnosis of patients with *ABCA4* retinopathy, which is becoming increasingly important for patient identification for recruitment to potential therapeutic interventions.

## Figures and Tables

**Table 1:** Genotype classification based on the ProgStar study.

Genotype Group	Description
A	Two or more severe/null variants
B	One severe/null variant and at least one missense or in-frame insertion/deletion
C	Two or more missense or in-frame insertion/deletion

**Table 2:** Clinical and phenotypic characteristics of patients with novel variants. This table shows summary data on patients’ demographics (sex, age, and ethnicity), clinical characteristics (self-reported age of onset and visual acuity), and electrodiagnostic test results. Electrodiagnostic test results were graded based on the criteria described by Lois et al. [20]. Novel variants are shown in bold. CF: counting fingers; NP: not performed.

ID	Genotype	Gender	Ethnicity	Age of Onset	Baseline VA OD OS		Age at Baseline	Follow-Up VA OD OS		Age at Follow-Up	Symptoms	Electrodiagnostic Testing
Patient 3	c.3095del, p.(Gly1032Glufs*50) c.3113C>T, p.(Ala1038Val)	F	European	Asymp (56)	0.00	−0.10	46	−0.18	0.02	59	Asymptomatic	NP
Patient 4	**c.58_66+1del** c.5882G>A, p.(Gly1961Glu)	M	South Asian	32	0.58	0.68	35	NP	NP	-	Blurred vision, photopsia	Normal rod and cone ERG (group 1)
Patient 6	**c.1979_1994delins21** c.4685T>C, p.(Ile1562Thr)	F	European	27	0.00	0.00	30	NP	NP	-	Delayed dark adaptation	NP
Patient 7	**c.4936G>T, p.(Asp1646Tyr)** c.5461-10T>C	M	European	40	1.06	1.00	49	NP	NP	-	Blurred vision	NP
Patient 8	**c.3607+5G>C** c.6079C>T, p.(Leu2027Phe)	M	European	40	1.36	CF	68	0.48	CF	76	Blurred vision, photophobia, delayed dark adaptation, abnormal colour vision	Absent PERG, relatively normal rod ERG and normal cone ERG (group 1)
Patient 9	c.2598_2599insTGGA, p.(Thr867Trpfs*18) c.4634G>A, p.(Ser1545Asn)	F	European	Asymp (33)	0.06	0.02	33	0.00	0.00	35	Asymptomatic	Normal PERG, rod ERG, cone ERG and EOG. mfERG with 2 rings with slightly smaller amplitudes. (Relatively normal)
Patient 10	**c.6278T>C, p.(Leu2093Pro)** c.5882G>A, p.(Gly1961Glu)	F	South Asian	32	0.84	0.7	49	NP	NP	-	Blurred vision	NP
Patient 12	**c.1855A>T, p.(Ile619Phe)** c.5882G>A, p.(Gly1961Glu)	F	South Asian	30	NP	NP	-	NP	NP	-	Blurred vision	NP

**Table 3:** Genetic testing results in patients with novel *ABCA4* variants. The genotypes of patients with novel variants are shown together with an analysis of the effect of the variant, their location along the *ABCA4* gene and protein, results of familial segregation studies, and genotype severity based on ProgStar criteria [19].

	Sex	Novel Variant	Protein	Mutation Effect	Position	Domain	Segregation	Other Variants	Protein	Position	Domain	Segregation	Genotype Severity
Patient 1	M	del exon 7	-	Exon deletion	Ex 7	ECD1	Affected Sister	Exon 7 del	-	Exon 7	ECD1	Affected Sister	A
Patient 2	F	c.365_366insCA	p.(Gly123fs*32)	Frameshift	Ex 4	ECD1	NP	c.634C>T	p.(Arg212Cys)	Exon 6	ECD1	NP	B
Patient 3	F	c.3095del	p.(Gly1032Glufs*50)	Frameshift	Ex 21	NBD1	NP	c.3113C>T	p.(Ala1038Val)	Exon 21	NBD1	NP	B
Patient 4	M	c.58_66+1del	-	Splicing	Int 1	TMD1	NP	c.5882G>A	p.(Gly1961Glu)	Exon 42	NBD2	NP	B
Patient 5	F	c.1100-2A>T	Splicing	Splicing	Int 8	ECD1	NP	c.3259G>A	p.(Glu1087Lys)	Exon 22	NBD1	NP	B
Patient 6	F	c.1979_1994delins21	p.(Val660fs)	Frameshift	Ex 14	TMD1	NP	c.4685T>C	p.(Ile1562Thr)	Exon 33	ECD2	NP	B
Patient 7	M	c.4936G>T	p.(Asp1646Tyr)	Missense	Ex 35	ECD2	NP	c.5461-10T>C	Splicing	Intron 38	TMD2	NP	B
Patient 8	M	c.3607+5G>C	Splicing	Splicing	Int 24	NBD1	NP	c.6079C>T	p.(Leu2027Phe)	Exon 44	NBD2	NP	B
Patient 9	F	c.2598_2599insTGGA	p.(Thr867Trpfs*18)	Frameshift	Ex 17	NBD1	Father	c.4634G>A	p.(Ser1545Asn)	Exon 31	ECD1	Mother	B
Patient 10	F	c.6278T>C	p.(Leu2093Pro)	Missense	Ex 45	NBD2	NP	c.5882G>A	p.(Gly1961Glu)	Exon 42	NBD2	NP	C
Patient 11	F	c.3607+5G>C	Splicing	Splicing	Int 24	NBD1	Mother	c.3210_3211dup	p.(Ser1071Cysfs*13)	Exon 22	NBD1	Father	A
Patient 12	F	c.1855A>T	p.(Ile619Phe)	Missense	Ex 13	ECD1	NP	c.5882G>A	p.(Gly1961Glu)	Exon 42	NBD2	NP	C

ECD: Exocytoplasmic domain; F: female; M: male; NBD: nucleotide-binding domain; NP: not performed; TMD: transmembrane domain.

**Table 4:** Pathogenicity analysis of novel *ABCA4* variants. This table summarises the results of in silico analysis and allele frequencies in publicly available databases for the novel variants that were detected in 12 patients. The table shows the results of the in silico analysis using three different programmes, SIFT, PolyPhen2 and MutationTaster. Allele frequencies in the gnomAD and ExAC databases of novel *ABCA4* variants detected in the Oxford *ABCA4* retinopathy cohort are shown, as well as the CADD score. ACMG: American College of Medical Genetics; CADD: combined annotation-dependent duplication; SIFT: Sorting Intolerant from Tolerance; NR: not reported; VUS: variant of uncertain significance.

	Novel Variant	Protein	CADD Score	Mutation Taster	SIFT	PolyPhen2	SpliceAI Score	ACMG	Allele Frequency GnomAD	Allele Frequency ExAC
Patient 1	del exon 7	-	-	-	-	-	-	Likely Pathogenic (PVS1_mod; PM2; PP4; PS4_sup)	-	-
Patient 2	c.365_366insCA	p.(Gly123fs*32)	27.8	-	-	-	0.00	Pathogenic (PVS1; PM2)	NR	NR
Patient 3	c.3095del	p.(Gly1032Glufs*50)	34	Disease causing	-	-	0.11	Pathogenic (PVS1; PM2)	NR	NR
Patient 4	c.58_66+1del	Splicing	33	-	-	-	1.00	Likely Pathogenic (PVS1_mod; PM2; PP4; PM3_sup)	NR	NR
Patient 5	c.1100-2A>T	Splicing	34	-	-	-	0.97	Pathogenic (PVS1; PM2)	NR	NR
Patient 6	c.1979_1994delins21	p.(Val660fs)	-	-	-	-	-	Pathogenic (PVS1; PM2)	NR	NR
Patient 7	c.4936G>T	p.(Asp1646Tyr)	24.3	Disease causing	Damaging	Benign	0.07	VUS (PM2; PM3; PP4)	NR	NR
Patient 8 & 11	c.3607+5G>C	Splicing	22.7	-	-	-	0.64	VUS (PM2; PM3; PP4)	NR	NR
Patient 9	c.2598_2599insTGGA	p.(Thr867Trpfs*18)	33	-	-	-	0.05	Pathogenic (PVS1; PM2)	NR	NR
Patient 10	c.6278T>C	p.(Leu2093Pro)	31	Disease causing	Damaging	Probably damaging	0.02	VUS (PM2; PP3; PP4)	NR	NR
Patient 12	c.1855A>T	p.(Ile619Phe)	23.2	Disease causing	Tolerated	Benign	0.02	VUS (PM2; PP3; PM3_sup)	0.0004%	NR

## Data Availability

Dataset available on request from the authors.

## Abbreviations

The following abbreviations are used in this manuscript:

FAF	Fundus autofluorescence
CADD	Combined annotation-dependent duplication
CNV	Copy Number Variant
ECD	Exocytoplasmic domain
EDT	Electrodiagnostic testing
MLPA	Multiplex ligation-dependent probe amplification
NGS	Next-Generation Sequencing
NBD	Nucleotide-binding domain
NMD	Nonsense-mediated decay
SD-OCT	Spectral-domain optical coherent tomography
Sm-MIPs	Single-molecule molecular inversion probes
Stargardt disease	STGD1
TMD	Transmembrane domain
SIFT	Sorting Intolerant from Tolerance
ISCEV	International Society for Clinical Electrophysiology of Vision
